# Multiple Positive Solutions for Nonlinear Fractional Boundary Value Problems

**DOI:** 10.1155/2013/473828

**Published:** 2013-11-06

**Authors:** Daliang Zhao, Yansheng Liu

**Affiliations:** School of Mathematical Sciences, Shandong Normal University, Jinan, Shandong 250014, China

## Abstract

This paper is devoted to the existence of multiple positive solutions for fractional boundary value problem DC0+α *u*(*t*) = *f*(*t*, *u*(*t*), *u*′(*t*)), 0 < *t* < 1, *u*(1) = *u*′(1) = *u*′′(0) = 0, where 2 < *α* ≤ 3 is a real number, DC0+α is the Caputo fractional derivative, and *f* : [0,1]×[0, +∞) × *R* → [0, +∞) is continuous. Firstly, by constructing a special cone, applying Guo-Krasnoselskii's fixed point theorem and Leggett-Williams fixed point theorem, some new existence criteria for fractional boundary value problem are established; secondly, by applying a new extension of Krasnoselskii's fixed point theorem, a sufficient condition is obtained for the existence of multiple positive solutions to the considered boundary value problem from its auxiliary problem. Finally, as applications, some illustrative examples are presented to support the main results.

## 1. Introduction

This paper investigates the existence of multiple positive solutions for the following nonlinear fractional boundary value problem (BVP, for short):

(1) DC0+αu(t)=f(t,u(t),u′(t)), 0<t<1,u(1)=u′(1)=u′′(0)=0,

where 2 < *α* ≤ 3 is a real number, DC0+α is the Caputo fractional derivative, and *f* : [0,1]×[0, +*∞*) × *R* → [0, +*∞*) is continuous.

Recently, fractional differential equations have gained considerable importance due to their wide applications [1–8] in various sciences such as mechanics, chemistry, physics, control theory, and engineering. Much attention has been focused on the solutions of differential equations of fractional order. Some kinds of methods are presented, such as the upper and lower method [9–11], the Laplace transform method [12, 13], the iteration method [14], the Fourier transform method [15], the homotopy analysis method [16, 17], and the Green function method [18–20]. In recent years, there are some papers dealing with the existence and multiplicity of solution to the nonlinear fractional BVP; for details, see [8, 9, 11, 21] and references therein.

In [21], Bai and Qiu investigated the following nonlinear fractional BVP:

(2) $$\begin{array}{c} D_{0^{+}}^{\alpha }u\left(t\right)+f\left(t,u\left(t\right)\right)=0,\;0<t<1,\\ u\left(0\right)=u^{\prime }\left(1\right)=u^{\prime \prime }\left(0\right)=0, \end{array}$$

where 2 < *α* ≤ 3 is a real number, $D_{0^{+}}^{\alpha }$ is the Caputo fractional derivative, and *f* : (0,1]×[0, *∞*)→[0, +*∞*) is continuous. By using Guo-Krasnoselskii's fixed point theorem and nonlinear alternative of Leray-Schauder, they established the existence and multiplicity of solutions to the above fractional BVP.

In [11], Zhao et al. established the existence of multiple positive solutions for the nonlinear fractional BVP:

(3) D0+αu(t)+f(t,u(t))=0, 0<t<1,        u(0)=u′(0)=u′(1)=0,

where 2 < *α* ≤ 3 is a real number, $D_{0^{+}}^{\alpha }$ is the Riemann-Liouville fractional derivative, and *f* ∈ *C*([0, 1], [0, *∞*)). The authors obtained the existence of positive solutions by the lower and upper solution method and fixed-point theorem.

In [8], Yang et al. investigated the existence of positive solutions of the BVP for differential equation of fractional order:

(4) $$\begin{array}{c} D_{0^{+}}^{\alpha }u\left(t\right)=f\left(t,u\left(t\right),u^{\prime }\left(t\right)\right),\;0<t<1,\\ u\left(0\right)+u^{\prime }\left(0\right)=0,\;\;u\left(1\right)+u^{\prime }\left(1\right)=0, \end{array}$$

where 1 < *α* ≤ 2 is a real number, $D_{0^{+}}^{\alpha }$ is the Caputo fractional derivative, and *f* : [0,1]×[0, *∞*) × *R* → [0, *∞*) is continuous. By means of a new fixed point theorem and Schauder fixed theorem, some results on the existence of positive solutions are obtained.

Though the fractional boundary value problems have been studied by lots of authors, there are few pieces of work considering the case that the nonlinear term *f* depends on the first order derivative *u*′(*t*). In addition, to the best of our knowledge, there is no paper discussing the existence of multiple positive solutions for BVP (1). By constructing a special cone, using Guo-Krasnoselskii and Leggett-Williams fixed point theorems, two sufficient conditions are established for the existence of multiple positive solutions to BVP (1). In addition, by virtue of a new extension of Krasnoselskii's fixed point theorem, a sufficient condition is obtained for the existence of multiple positive solutions of BVP (1) from its auxiliary problem. Finally, some illustrative examples are worked out to demonstrate the main results.

The organization of this paper is as follows.  Section 2 contains some definitions and lemmas of fractional calculus theory which will be used in the next two sections. In Section 3, we establish the existence results on multiple positive solutions to BVP (1) by Guo-Krasnoselskii, Leggett-Williams fixed point theorem, and another new extension of Krasnoselskii's fixed point theorem. Finally, some examples are presented to support the obtained results in Section 4. 

## 2. Preliminary Results

In this section, we introduce some necessary definitions and preliminary facts which will be used throughout this paper.

**Definition (see [15])**

The Caputo fractional derivative of order *α* > 0 of a continuous function *u* : (0, *∞*) → *R* is given by

(5) DC0+αu(t)=1Γ(n−α)∫0tu(n)(s)(t−s)α−n+1ds,

where *n* = [*α*] + 1, [*α*] denotes the integer part of the real number *α* and provided that the right side integral is pointwise defined on [0, *∞*).

**Definition (see [15])**

The Riemann-Liouville standard fractional derivative of order *β* > 0 of a continuous function *ν* : (0, *∞*) → *R* is given by

(6) $$\begin{array}{c} D_{0^{+}}^{\beta }u\left(t\right)=\frac{1}{\Gamma \left(n-\beta \right)}{\left(\frac{d}{dt}\right)}^{n}\overset{t}{\underset{0}{\int }}\frac{\nu \left(s\right)}{{\left(t-s\right)}^{\beta -n+1}}ds, \end{array}$$

where *n* = [*β*] + 1, [*β*] denotes the integer part of the real number *β*, and provided that the right side integral is pointwise defined on [0, *∞*).

**Definition (see [15])**

The Riemann-Liouville standard fractional integral of order *α* > 0 of a continuous function *u* : (0, *∞*) → *R* is given by

(7) $$\begin{array}{c} I_{0^{+}}^{\alpha }u\left(t\right)=\frac{1}{\Gamma \left(\alpha \right)}\overset{t}{\underset{0}{\int }}{\left(t-s\right)}^{\alpha -1}u\left(s\right)ds, \end{array}$$

provided that the right side integral is pointwise defined on [0, *∞*).

**Lemma 4 (see [15])**

Let *n* − 1 < *α* ≤ *n*  (*n* ∈ *N*). Then,

(8) I0+αDC0+αu(t)=u(t)+c0+c1t+c2t2+⋯+cn−1tn−1,

for some *c*_*i*_ ∈ *R*, *i* = 0,1,…, *n* − 1, *n* = [*α*] + 1.

**Lemma (see [15])**

Let *α* > 0 and *y* ∈ *C*[*a*, *b*]. Then,

(9) (DCa+αIa+αy)(x)=y(x)

holds on [*a*, *b*].

**Lemma 6 (see [15])**

Let *α* > *β* > 0. If one assumes that *u*(*t*) ∈ *C*(0,1)∩*L*(0,1), then $D_{0^{+}}^{\beta }I_{0^{+}}^{\alpha }$ *u*(*t*) = $I_{0^{+}}^{\alpha -\beta }$ *u*(*t*).

**Lemma 7 (see [15])**

Let *n* − 1 < *α* ≤ *n*  (*n* ∈ *N*). The fractional differential equation $D_{0^{+}}^{\alpha }$ *x*(*t*) = 0 has solution

(10) $$\begin{array}{c} x\left(t\right)=c_{0}+c_{1}t+c_{2}t^{2}+\cdots +c_{n-1}t^{n-1}, \end{array}$$

for some *c*_*i*_ ∈ *R*, *i* = 0,1,…, *n* − 1, *n* = [*α*] + 1.

**Lemma 8**

For any *g* ∈ *C*[0,1] and 2 < *α* ≤ 3, the unique solution of problem

(11) DC0+αu(t)=g(t), 0<t<1;u(1)=u′(1)=u′′(0)=0,

is

(12) $$\begin{array}{c} u\left(t\right)=\overset{1}{\underset{0}{\int }}G\left(t,s\right)g\left(s\right)ds,\;t\in \left[0,1\right], \end{array}$$

where

(13) $$\begin{array}{c} G\left(t,s\right)=\{\begin{array}{cc} \frac{{\left(t-s\right)}^{\alpha -1}-{\left(1-s\right)}^{\alpha -1}}{\Gamma \left(\alpha \right)} & \\ \;+\frac{\left(1-t\right){\left(1-s\right)}^{\alpha -2}}{\Gamma \left(\alpha -1\right)} & 0\leq s\leq t\leq 1,\\ \frac{\left(1-t\right){\left(1-s\right)}^{\alpha -2}}{\Gamma \left(\alpha -1\right)} & \\ \;-\frac{{\left(1-s\right)}^{\alpha -1}}{\Gamma \left(\alpha \right)} & 0\leq t\leq s\leq 1. \end{array} \end{array}$$

Here, *G*(*t*, *s*) is said to be the Green function of BVP (11).

**Proof**

In view of Lemma 4, (11) is equivalent to the integral equation

(14) $$\begin{array}{c} u\left(t\right)=I_{0^{+}}^{\alpha }g\left(t\right)+c_{0}+c_{1}t+c_{2}t^{2}, \end{array}$$

for some *c*_*i*_ ∈ *R*, *i* = 0,1, 2. So, we have

(15) $$\begin{array}{c} u^{\prime }\left(t\right)=I_{0^{+}}^{\alpha -1}g\left(t\right)+c_{1}+2c_{2}t,\\ u^{\prime \prime }\left(t\right)=I_{0^{+}}^{\alpha -2}g\left(t\right)+2c_{2}. \end{array}$$

From the boundary condition *u*(1) = *u*′(1) = *u*′′(0) = 0, one has

(16) $$\begin{array}{c} c_{1}=-I_{0^{+}}^{\alpha -1}g\left(t\right),\;\;c_{2}=0,\;\;c_{3}=I_{0^{+}}^{\alpha -1}g\left(t\right)-I_{0^{+}}^{\alpha }g\left(1\right). \end{array}$$

Therefore, by Definition 3, we conclude that the unique solution of BVP (11) is

(17) u(t) =∫0t[(t−s)α−1−(1−s)α−1Γ(α)+(1−t)(1−s)α−2Γ(α−1)]g(s)ds  +∫t1[(1−t)(1−s)α−2Γ(α−1)−(1−s)α−1Γ(α)]g(s)ds =∫01G(t,s)g(s)ds.

The proof is completed.

The following properties of the Green function play an important role in this paper.

**Lemma 9**

Green function *G*(*t*, *s*) defined as (13) satisfies the following conditions.

(i) *G*(*t*, *s*) ∈ *C*([0,1]×[0,1]) and *G*(*t*, *s*) > 0 for any *t*, *s* ∈ [0,1).
(ii) There exist a positive number *η* and a positive function *γ*(*s*) ∈ *C*[0,1) such that
  (18) $$\begin{array}{c} \underset{t\in \left[0,\eta \right]}{\min}G\left(t,s\right)\geq \eta \gamma \left(s\right),\;\underset{t\in \left[0,1\right]}{\max}G\left(t,s\right)\leq \gamma \left(s\right),\;s\in \left[0,1\right). \end{array}$$

**Proof**

(i) It is obvious that *G*(*t*, *s*) is continuous on [0,1]×[0,1]. For 0 ≤ *s* ≤ *t* < 1, we have

(19) ∂G∂t=(α−1)(t−s)α−2Γ(α)−(1−s)α−2Γ(α−1)=(t−s)α−2−(1−s)α−2Γ(α−1)<0.

Similarly, we can obtain that

(20) $$\begin{array}{c} \frac{\partial G}{\partial t}=\frac{-{\left(1-s\right)}^{\alpha -2}}{\Gamma \left(\alpha -1\right)}<0,\;0\leq t\leq s<1. \end{array}$$

Hence, ∂*G*/∂*t* < 0 for all *s*, *t* ∈ [0,1). In addition, it is clear that *G*(1, *s*) = 0 for 0 ≤ *s* < 1. Therefore, we get that *G*(*t*, *s*) > 0 for any *t*, *s* ∈ [0,1).

(ii) In the following, we consider the existence of *η* and *γ*(*s*).

Firstly, if 0 ≤ *s* ≤ *t* < 1, then by the definition of *G*(*t*, *s*), we have

(21) G(t,s)≥−(1−s)α−1Γ(α)+(1−t)(1−s)α−2Γ(α−1)≥−(1−s)α−2Γ(α)+(1−t)(1−s)α−2Γ(α−1)=(1−s)α−2Γ(α−1)(α−2α−1−t).

If 0 ≤ *t* ≤ *s* < 1, then by an argument similar to the case 0 ≤ *s* ≤ *t* < 1, we also have *G*(*t*, *s*) ≥ ((1 − *s*)^*α*−2^/Γ(*α* − 1))((*α* − 2)/(*α* − 1) − *t*).

Secondly, for given 0 ≤ *s* ≤ *t* < 1, it is obvious that

(22) $$\begin{array}{c} G\left(t,s\right)\leq \frac{\left(1-t\right){\left(1-s\right)}^{\alpha -2}}{\Gamma \left(\alpha -1\right)}\leq \frac{{\left(1-s\right)}^{\alpha -2}}{\Gamma \left(\alpha -1\right)}. \end{array}$$

As 0 ≤ *t* ≤ *s* < 1, we also have that

(23) $$\begin{array}{c} G\left(t,s\right)\leq \frac{\left(1-t\right){\left(1-s\right)}^{\alpha -2}}{\Gamma \left(\alpha -1\right)}\leq \frac{{\left(1-s\right)}^{\alpha -2}}{\Gamma \left(\alpha -1\right)}. \end{array}$$

Thus, setting

(24) $$\begin{array}{c} \eta =\frac{\alpha -2}{2\left(\alpha -1\right)},\;\;\gamma \left(s\right)=\frac{{\left(1-\text{s}\right)}^{\alpha -2}}{\Gamma \left(\alpha -1\right)}, \end{array}$$

we immediately obtain that

(25) $$\begin{array}{c} \underset{t\in \left[0,\eta \right]}{\min}G\left(t,s\right)\geq \eta \gamma \left(s\right),\;\underset{t\in \left[0,1\right]}{\max}G\left(t,s\right)\leq \gamma \left(s\right),\;s\in \left[0,1\right). \end{array}$$

The proof is completed.

Now, we list the following fixed point theorems which will be used in the next section.

**Lemma 10 (see [22], (Guo-Krasnoselskii's fixed point theorem))**

Let *E* be a Banach space, *P*⊆*E* a cone, and *Ω*_1_, *Ω*_2_ two bounded open balls of *E* centered at the origin with ${\bar{\Omega }}_{1}\subset \Omega_{2}$. Suppose that $A:P\cap ({\bar{\Omega }}_{2}\setminus \Omega_{1})\to P$ is a completely continuous operator such that either

- ||*Ax*|| ≤ ||*x*||, *x* ∈ *P*∩∂*Ω*_1_ and ||*Ax*|| ≥ ||*x*||, *x* ∈ *P*∩∂*Ω*_2_, or
- ||*Ax*|| ≥ ||*x*||, *x* ∈ *P*∩∂*Ω*_1_ and ||*Ax*|| ≤ ||*x*||, *x* ∈ *P*∩∂*Ω*_2_,

holds. Then, *A* has a fixed point in $P\cap ({\bar{\Omega }}_{2}\setminus \Omega_{1})$.

For the sake of stating Leggett-Williams fixed point theorem, we first give the definition of concave functions.

**Definition (see [11])**

The map *θ* is said to be a nonnegative concave functional on a cone *P* of a real Banach space *E* provided that *θ* : *P* → [0, *∞*) is continuous and

(26) $$\begin{array}{c} \theta \left(tx+\left(1-t\right)y\right)\geq t\theta \left(x\right)+\left(1-t\right)\theta \left(y\right), \end{array}$$

for all *x*, *y* ∈ *P* and 0 ≤ *t* ≤ 1.

**Lemma 12 (see [23], (Leggett-Williams fixed point theorem))**

Let *P* be a cone in a real Banach space *E*, *P*_*c*_ = {*x* ∈ *P* : ||*x*|| ≤ *c*}, *θ* a nonnegative continuous concave functional on *P* such that *θ*(*x*) ≤ ||*x*|| for all $x\in {\bar{P}}_{c}$, and *P*(*θ*, *b*, *d*) = {*x* ∈ *P* : *b* ∈ *θ*(*x*), ||*x*|| ≤ *d*}. Suppose that $A:{\bar{P}}_{c}\to {\bar{P}}_{c}$ is completely continuous and there exist constants 0 < *a* < *b* < *d* ≤ *c* such that

(*C*_1_) {*x* ∈ *P*(*θ*, *b*, *d*) : *θ*(*x*) > *b*} ≠ *∅* and *θ*(*Ax*) > *b* for *x* ∈ *P*(*θ*, *b*, *d*);
(*C*_2_) ||*Ax*|| < *a* for *x* ≤ *a*;
(*C*_3_) *θ*(*Ax*) > *b* for *x* ∈ *P*(*θ*, *b*, *c*) with ||*Ax*|| > *d*.

Then, *A* has at least three fixed points *x*_1_, *x*_2_, and *x*_3_ with ||*x*_1_|| < *a*, *b* < *θ*(*x*_2_), *a* < ||*x*_3_|| with *θ*(*x*_3_) < *b*.

**Remark**

If *d* = *c* holds, then condition (*C*_1_) of Lemma 12 implies condition (*C*_3_).

Finally, in this section, we give a new extension of Krasnoselskii's fixed point theorem, which is developed in [24].

Let *X* be a Banach space and *P* ⊂ *X* a cone. Suppose that *α*, *β* : *X* → *R* ^+^ are two continuous convex functions satisfying

(27) $$\begin{array}{c} \alpha \left(\lambda u\right)=\left|\lambda \right|\alpha \left(u\right),\;\;\;\beta \left(\lambda u\right)=\left|\lambda \right|\beta \left(u\right) \end{array}$$

for *u* ∈ *X*, *λ* ∈ *R*; ||*u*|| ≤ *ϱ*max⁡{*α*(*u*), *β*(*u*)} for *u* ∈ *X*; and *α*(*u*_1_) ≤ *α*(*u*_2_) for *u*_1_, *u*_2_ ∈ *P*, *u*_1_ ≤ *u*_2_, where *ϱ* > 0 is a constant.

**Lemma 14 (see [24])**

Let *r*_2_ > *r*_1_ > 0, *L* > 0 be constants and *Ω*_*i*_ = {*u* ∈ *X* : *α*(*u*) < *r*_*i*_, *β*(*u*) < *L*}, (*i* = 1,2) two bounded open sets in *X*. Set *D*_*i*_ = {*u* ∈ *X* : *α*(*u*) = *r*_*i*_}. Assume that *T* : *P* → *P* is a completely continuous operator satisfying

(*D*_1_) *α*(*Tu*) < *r*_1_, *u* ∈ *D*_1_∩*P*; *α*(*Tu*) > *r*_2_, *u* ∈ *D*_2_∩*P*;
(*D*_2_) *β*(*Tu*) < *L*, *u* ∈ *P*;
(*D*_3_) there is a *p* ∈ (*Ω*_2_∩*P*)∖{0} such that *α*(*p*) ≠ 0 and *α*(*u* + *λp*) ≥ *α*(*u*) for all *u* ∈ *P* and *λ* ≥ 0.

Then, *T* has at least one fixed point in $(\Omega_{2}\setminus {\bar{\Omega }}_{1})\cap P$.

## 3. Main Results

In this section, we assume that *f* : [0,1]×[0, +*∞*) × *R* → [0, +*∞*) is continuous and satisfies some specific growth conditions, which allows us to apply Lemmas 10–14 to establish the existence of multiple positive solutions for BVP (1).

First, let *X* = *C* ^1^[0,1] be endowed with the norm

(28) $$\begin{array}{c} ||u||=\underset{t\in \left[0,1\right]}{\max}\left|u\left(t\right)\right|+\underset{t\in \left[0,1\right]}{\max}\left|u^{\prime }\left(t\right)\right|={||u||}_{0}+{||u^{\prime }||}_{0}. \end{array}$$

Define the set *P* ⊂ *X* by

(29) $$\begin{array}{c} P=\left\{u\in X:u\left(t\right)\geq 0;\;u\left(1\right)=0;\;u\left(t\right)\geq \eta {||u||}_{0},t\in \left[0,\eta \right]\right\}. \end{array}$$

It is easy to verify that *P* is a cone in the space *X*.

Let the nonnegative continuous concave function *θ* on the cone *P* be defined by

(30) $$\begin{array}{c} \theta \left(u\right)=\underset{t\in \left[0,\eta \right]}{\min}\left|u\left(t\right)\right|. \end{array}$$

Define an operator *T* on *P* by the formula

(31) $$\begin{array}{c} \left(Tu\right)\left(t\right)=\overset{1}{\underset{0}{\int }}G\left(t,s\right)f\left(s,u\left(s\right),u^{\prime }\left(s\right)\right)ds. \end{array}$$

**Lemma 15**

The operator *T* : *P* → *P* is completely continuous.

**Proof**

For any *u* ∈ *P*, we have that *Tu*(*t*) ≥ 0 in view of nonnegativeness of *G*(*t*, *s*) and *f*(*s*, *u*, *u*′). It is obvious that

(32) $$\begin{array}{c} \left(Tu\right)\left(1\right)=\overset{1}{\underset{0}{\int }}G\left(1,s\right)f\left(s,u\left(s\right),u^{\prime }\left(s\right)\right)ds=0. \end{array}$$

By Lemma 9, for any *t* ∈ [0, *η*] and *τ* ∈ [0,1], we obtain that

(33) (Tu)(t)≥η∫01γ(s)f(s,u,u′)ds≥η∫01G(τ,s)f(s,u,u′)ds=η(Tu)(τ)≥η||Tu||0.

Hence, *T*(*P*) ⊂ *P*.

The operator *T* : *P* → *P* is continuous in view of continuity of *G*(*t*, *s*) and *f*(*s*, *u*, *u*′).

Let *U* ⊂ *P* be bounded; that is, there exists a positive constant *K* > 0 such that ||*u*|| ≤ *K* for all *u* ∈ *U*. By means of the definition of ||*u*||, we have |*u*(*t*)| ≤ *K* and |*u*′(*t*)| ≤ *K* for *t* ∈ [0, 1]. Let *C* = max⁡_*t*∈[0,1],*u*∈*U*_⁡|*f*(*t*, *u*(*t*), *u*′(*t*))| + 1. Then, for *u* ∈ *U*, by Lemma 9, we have

(34) |(Tu)(t)|≤∫01G(t,s)f(s,u(s),u′(s))ds≤C∫01γ(s)ds≤2CΓ(α),|(Tu′)(t)|≤C[1Γ(α−1)∫0t(t−s)α−2ds   +1Γ(α−1)∫01(1−s)α−2ds]≤2CΓ(α).

Hence, *T*(*U*) is bounded.

For each *u* ∈ *U*, *t*_1_, *t*_2_ ∈ [0,1] with *t*_1_ < *t*_2_, then

(35) |(Tu)(t2)−(Tu)(t1)| ≤∫0t1|[G(t2,s)−G(t1,s)]f(s,u,u′)|ds  +∫t21|[G(t2,s)−G(t1,s)]f(s,u,u′)|ds  +∫t1t2|[G(t2,s)−G(t1,s)]f(s,u,u′)|ds ≤C{∫0t1[(t2−s)α−1−(t1−s)α−1Γ(α)           +(1−s)α−2(t2−t1)Γ(α−1)]ds        +∫t21(1−s)α−2(t2−t1)Γ(α−1)ds        +∫t1t2[(t2−s)α−1Γ(α)+(1−s)α−2(t2−t1)Γ(α−1)]ds} ≤C[t2−t1Γ(α−1)+α−1Γ(α)(t2−t1)+(t2−t1)αΓ(α)] =C[2(t2−t1)Γ(α−1)+(t2−t1)αΓ(α)],|(Tu)′(t2)−(Tu)′(t1)| =|∫0t2(t2−s)α−2Γ(α−1)f(s,u,u′)ds   −∫0t1(t1−s)α−2Γ(α−1)f(s,u,u′)ds| ≤C|∫0t1(t2−s)α−2−(t1−s)α−2Γ(α−1)ds+∫t1t2(t2−s)α−2Γ(α−1)ds| ≤C[t2α−1−t1α−1Γ(α)+(t2−t1)α−1Γ(α)+(t2−t1)α−1Γ(α)] ≤CΓ(α)[2(t2−t1)α−1+(t2α−1−t1α−1)].

By means of the Arzela-Ascoli theorem, one can obtain that the operator *T* is completely continuous. The proof is completed.

Now, we are in a position to state the main results.

For convenience, denote

(36) $$\begin{array}{c} M={\left(\overset{1}{\underset{0}{\int }}3\gamma \left(s\right)ds\right)}^{-1},\;\;N={\left(\overset{\eta }{\underset{0}{\int }}\eta \gamma \left(s\right)ds\right)}^{-1}. \end{array}$$

**Theorem 16**

Assume that there exist two positive constants *σ*_2_ > *σ*_1_ > 0 such that

(*H*_1_) *f*(*t*, *u*, *v*) ≤ *Mσ*_2_ for (*t*, *u*, *v*)∈[0,1]×[0, *σ*_2_]×[−*σ*_2_, *σ*_2_];
(*H*_2_) *f*(*t*, *u*, *v*) ≥ *Nσ*_1_ for (*t*, *u*, *v*)∈[0,1]×[0, *σ*_1_]×[−*σ*_1_, *σ*_1_].

Then, BVP (1) has at least one solution *u* such that *σ*_1_ ≤ ||*u*|| ≤ *σ*_2_.

**Proof**

By Lemma 15, we know that the operator *T* : *P* → *P* defined by (31) is completely continuous.

(i) Let *Ω*_2_ = {*u* ∈ *P* : ||*u*|| < *σ*_2_}. For any *u* ∈ *P*∩∂*Ω*_2_, we have 0 ≤ *u*(*t*) ≤ *σ*_2_, −*σ*_2_ ≤ *u*′(*t*) ≤ *σ*_2_ for all *t* ∈ [0,1]. It follows from condition (*H*_1_) and Lemma 9 that, for *t* ∈ [0,1],

(37) ||Tu||=max⁡t∈[0,1]|∫01G(t,s)f(s,u,u′)ds| +max⁡t∈[0,1]|∫01∂G(t,s)∂tf(s,u,u′)ds|≤Mσ2[∫01γ(s)ds+∫012γ(s)ds]=Mσ2∫013γ(s)ds=σ2=||u||,

which implies that ||*Tu*|| ≤ ||*u*||, *u* ∈ *P*∩∂*Ω*_2_.

(ii) Let *Ω*_1_ = {*u* ∈ *P* : ||*u*|| < *σ*_1_}. For any *u* ∈ *P*∩∂*Ω*_1_, we have 0 ≤ *u*(*t*) ≤ *σ*_1_, −*σ*_1_ ≤ *u*′(*t*) ≤ *σ*_1_ for all *t* ∈ [0,1]. It follows from condition (*H*_2_) and Lemma 9 that, for *t* ∈ [0,1],

(38) ||Tu||=max⁡t∈[0,1]|∫01G(t,s)f(s,u,u′)ds| +max⁡t∈[0,1]|∫01∂G(t,s)∂tf(s,u,u′)ds|≥max⁡t∈[0,η]|∫01G(t,s)f(s,u,u′)ds|≥Nσ1[η∫0ηγ(s)ds]=σ1=||u||,

which implies that ||*Tu*|| ≥ ||*u*||, *u* ∈ *P*∩∂*Ω*_1_.

In view of Lemma 10, *T* has a fixed point in $P\cap ({\bar{\Omega }}_{2}\setminus \Omega_{1})$ which is a solution of BVP (1). The proof is completed.

**Theorem 17**

Suppose that there exist constants 0 < *a* < *b* < *c* such that the following assumptions hold:

(*A*_1_) *f*(*t*, *u*, *v*) < *Ma* for (*t*, *u*, *v*)∈[0,1]×[0, *a*]×[−*a*, *a*];
(*A*_2_) *f*(*t*, *u*, *v*) ≥ *Nb* for (*t*, *u*, *v*)∈[0, *η*]×[*b*, *c*]×[−*c*, *c*];
(*A*_3_) *f*(*t*, *u*, *v*) ≤ *Mc* for (*t*, *u*, *v*)∈[0,1]×[0, *c*]×[−*c*, *c*].

Then, BVP (1) has at least three positive solutions *u*_1_, *u*_2_, and *u*_3_ with

(39) $$\begin{array}{c} \underset{t\in \left[0,1\right]}{\max}\left|u_{1}\left(t\right)\right|<a,\;\;b<\underset{t\in [0,\eta ]}{\min}\left|u_{2}\left(t\right)\right|<\underset{t\in \left[0,1\right]}{\max}\left|u_{2}\left(t\right)\right|\leq c,\\ a<\underset{t\in \left[0,1\right]}{\max}\left|u_{3}\left(t\right)\right|\leq c,\;\;\underset{t\in [0,\eta ]}{\min}\left|u_{3}\left(t\right)\right|<b. \end{array}$$

**Proof**

We will show that all the conditions of Lemma 12 are satisfied.

First, if $u\in {\bar{P}}_{c}$, then ||*u*|| ≤ *c*. By condition (*A*_3_) and Lemma 9, we have

(40) ||Tu||=max⁡t∈[0,1]|∫01G(t,s)f(s,u,u′)ds| +max⁡t∈[0,1]|∫01∂G(t,s)∂tf(s,u,u′)ds|≤Mc[∫01γ(s)ds+∫012γ(s)ds]=Mc∫013γ(s)ds=c,

which implies that ||*Tu*|| ≤ *c* for $u\in {\bar{P}}_{c}$. Hence, $T:{\bar{P}}_{c}\to {\bar{P}}_{c}$.

Next, by using the analogous argument, it follows from condition (*A*_1_) that ||*Tu*|| < *a* for $u\in {\bar{P}}_{a}$.

Choose *u*(*t*) = (*b* + *c*)/2 for *t* ∈ [0,1]. It is easy to see that

(41) $$\begin{array}{c} u\left(t\right)=\frac{b+c}{2}\in P\left(\theta ,b,c\right),\;\;\theta \left(u\right)=\theta \left(\frac{b+c}{2}\right)>b; \end{array}$$

consequently, {*u* ∈ *P*(*θ*, *b*, *c*) | *θ*(*u*) > *b*} ≠ *∅*. Hence, if *u* ∈ *P*(*θ*, *b*, *c*), then *b* ≤ *u*(*t*) ≤ *c* for *t* ∈ [0, *η*]. By condition (*A*_2_), we have *f*(*t*, *u*(*t*), *u*′(*t*)) ≥ *Nb* for *t* ∈ [0, *η*]. So,

(42) θ(Tu)=min⁡t∈[0,η]|(Tu)(t)|=min⁡t∈[0,η]|∫01G(t,s)f(s,u(s),u′(s))ds|≥∫01ηγ(s)f(s,u(s),u′(s))ds≥N∫0ηηγ(s)ds·b=b,

which implies that *θ*(*Tu*) > *b* for *u* ∈ *P*(*θ*, *b*, *c*).

By Lemma 12, BVP (1) has at least three positive solutions *u*_1_, *u*_2_, and *u*_3_ with

(43) $$\begin{array}{c} \underset{t\in \left[0,1\right]}{\max}\left|u_{1}\left(t\right)\right|<a,\;\;\;b<\underset{t\in [0,\eta ]}{\min}\left|u_{2}\left(t\right)\right|<\underset{t\in \left[0,1\right]}{\max}\left|u_{2}\left(t\right)\right|\leq c,\\ a<\underset{t\in \left[0,1\right]}{\max}\left|u_{3}\left(t\right)\right|\leq c,\;\;\;\underset{t\in [0,\eta ]}{\min}\left|u_{3}\left(t\right)\right|<b. \end{array}$$

The proof is completed.

**Theorem 18**

Assume that there exist constants *L* > *d* > *ηd* > *q* > 0 such that

(*B*_1_) *f*(*t*, *u*, *v*) < *q*/*M*′ for (*t*, *u*, *v*)∈[0,1]×[0, *q*]×[−*L*, *L*];
(*B*_2_) *f*(*t*, *u*, *v*) ≥ *d*/*N*′ for (*t*, *u*, *v*)∈[0, *η*]×[*ηd*, *d*]×[−*L*, *L*];
(*B*_3_) *f*(*t*, *u*, *v*) < *L*/*Q* for (*t*, *u*, *v*)∈[0,1]×[0, *d*]×[−*L*, *L*],

where

(44) $$\begin{array}{c} M^{\prime }=\overset{1}{\underset{0}{\int }}\gamma \left(s\right)ds,\;\;N^{\prime }=\overset{\eta }{\underset{0}{\int }}\eta \gamma \left(s\right)ds,\;\;\;Q=\frac{2}{\Gamma \left(\alpha \right)}. \end{array}$$

Then, BVP (1) has at least one positive solution *u*(*t*) satisfying *q* < *α*(*u*) < *d*, |*u*′(*t*)| < *L* for *t* ∈ [0,1].

**Proof**

In order to apply the new extension of Krasnoselskii's fixed point theorem, we consider the following auxiliary BVP:

(45) D0+αCu(t)=f∗(t,u(t),u′(t)), 0<t<1,u(1)=u′(1)=u′′(0)=0,

where

(46) f∗(t,u,v)={f(t,u,v) (t,u,v)∈[0,1]×[0,d]×[−L,L],f(t,d,v) (t,u,v)∈[0,1]×(d,+∞)×[−L,L],f(t,u,−L) (t,u,v)∈[0,1]×[0,d]×(−∞,−L],f(t,d,−L) (t,u,v)∈[0,1]×(d,+∞)×(−∞,−L],f(t,u,L) (t,u,v)∈[0,1]×[0,d]×[L,+∞],f(t,d,L) (t,u,v)∈[0,1]×(d,+∞)×[L,+∞).

It is obvious that f* : [0,1]×[0, +*∞*) × *R* → [0, +*∞*) is continuous according to the continuity of *f*. By using the similar proof of Lemma 15, one can obtain that the operator T* given by

(47) $$\begin{array}{c} \left(T^{*}u\right)\left(t\right)=\overset{1}{\underset{0}{\int }}G\left(t,s\right)f^{*}\left(s,u\left(s\right),u^{\prime }\left(s\right)\right)ds \end{array}$$

is also completely continuous on *P* and maps *P* into *P*. Let

(48) $$\begin{array}{c} \Lambda_{1}=\left\{u\in X:\left|u\left(t\right)\right|<q,\left|u^{\prime }\left(t\right)\right|<L\right\},\\ \Lambda_{2}=\left\{u\in X:\left|u\left(t\right)\right|<d,\left|u^{\prime }\left(t\right)\right|<L\right\},\\ D_{1}=\left\{u\in X:\alpha \left(u\right)=q\right\},\;\;D_{2}=\left\{u\in X:\alpha \left(u\right)=d\right\}. \end{array}$$

It is obvious that there exists a nonnegative function *p* ∈ (Λ_2_∩*P*)∖{0} such that *α*(*u* + *λp*) ≥ *α*(*u*) for all *u* ∈ *P*, *λ* ≥ 0. We divide the proof into the following three steps.

*Step 1.* By virtue of condition (*B*_1_) and *α*(*u*) = *q*, *u* ∈ *D*_1_∩*P*, we have

(49) α(T∗u)=max⁡t∈[0,1]|∫01G(t,s)f∗(s,u(s),u′(s))ds|<max⁡t∈[0,1]|∫01G(t,s)qM′ds|≤qM′∫01γ(s)ds=q.

*Step 2.* For *α*(*u*) = *d*, *u* ∈ *D*_2_∩*P*, it follows from *u*(*t*) ≥ *η*||*u*||_0_, *t* ∈ [0, *η*], and condition (*B*_2_) that

(50) α(T∗u)=max⁡t∈[0,1]|∫01G(t,s)f∗(s,u(s),u′(s))ds|>max⁡t∈[0,1]|∫0ηG(t,s)dN′ds|≥dN′∫0ηηγ(s)ds=d.

*Step 3.* In view of condition (*B*_3_), for *u* ∈ *P*∩Λ_2_, we have

(51) β(T∗u) =max⁡t∈[0,1]|∫01∂G(t,s)∂tf∗(s,u(s),u′(s))ds| =max⁡t∈[0,1]|∫0t(t−s)α−2Γ(α−1)f∗(s,u,u′)ds         −∫01(1−s)α−2Γ(α−1)f∗(s,u,u′)ds| <LQ[1Γ(α−1)∫01(t−s)α−2ds        +1Γ(α−1)∫01(1−s)α−2ds] ≤LQ2Γ(α)=L.

Hence, *β*(T*u) < *L*. By Lemma 14, there exists $u\in (\Lambda_{2}\setminus {\bar{\Lambda }}_{1})\cap P$ such that *u*(*t*) = (T*u)(*t*). Consequently, *u*(*t*) is a positive solution for the auxiliary BVP (45) satisfying *q* < *α*(*u*) < *d*, |*u*′(*t*)| < *L*. In addition, by virtue of the definition of f*, we know that f*(*t*, *u*(*t*), *u*′(*t*)) = *f*(*t*, *u*(*t*), *u*′(*t*)), *t* ∈ [0,1]. Therefore, *u* is a positive solution of BVP (1). The proof is completed.

## 4. Examples

**Example 1**

Consider the following fractional BVP:

(52) D0+5/2Cu(t)=u2+(u′)22+et100, 0<t<1,u(1)=u′(1)=u′′(0)=0.

By a simple calculation, one can obtain that *η* = 1/6, $M=\sqrt{\pi }/4\approx 0.443$, and *N* ≈ 33.333. Choosing *σ*_1_ = 1/10^4^, *σ*_2_ = 1/10, we have

(53) $$\begin{array}{c} f\left(t,u,u^{\prime }\right)=\frac{u^{2}+{\left(u^{\prime }\right)}^{2}}{2}+\frac{e^{t}}{100}\leq 0.037\leq M\sigma_{2}\approx 0.0443 \end{array}$$

for (*t*, *u*, *u*′) ∈ [0,1]×[0, 1/10]×[−(1/10), 1/10] and

(54) $$\begin{array}{c} f\left(t,u,u^{\prime }\right)=\frac{u^{2}+{\left(u^{\prime }\right)}^{2}}{2}+\frac{e^{t}}{100}\geq 0.01\geq N\sigma_{1}\approx 0.00333 \end{array}$$

for (*t*, *u*, *u*′) ∈ [0,1] × [0, 1/10^4^]×[−(1/10^4^), 1/10^4^].

With the use of Theorem 16, BVP (52) has at least one solution *u* such that 1/10^4^ ≤ ||*u*|| ≤ 1/10.

**Example 2**

Consider the following fractional BVP:

(55) D0+5/2Cu(t)=f(t,u(t),u′(t)), 0<t<1,u(1)=u′(1)=u′′(0)=0,

where

(56) $$\begin{array}{c} f\left(t,u,v\right)=\{\begin{array}{cc} \frac{\sin\left(\pi t\right)}{10^{3}}+u^{4}+\frac{\sqrt{\left|v\right|}}{100}, & 0\leq u\leq 1,\\ 1+\frac{\sin\left(\pi t\right)}{10^{3}}+\frac{u}{20}+\frac{\sqrt{\left|v\right|}}{100}, & u>1. \end{array} \end{array}$$

Choosing *a* = 1/10, *b* = 1/50, and *c* = 4, then, there hold

(57) f(t,u,u′)=sin(πt)103+u4+|v|100≤0.0021<Ma≈0.00443

for (*t*, *u*, *u*′) ∈ [0,1]×[0, 1/100]×[−(1/100), 1/100]

(58) $$\begin{array}{c} f\left(t,u,u^{\prime }\right)=1+\frac{\sin\left(\pi t\right)}{10^{3}}+\frac{u}{20}+\frac{\sqrt{\left|v\right|}}{100}\geq 35\geq Nb\approx 33.333 \end{array}$$

for (*t*, *u*, *u*′) ∈ [0, 1/6]×[1,4]×[−4,4]; and

(59) f(t,u,u′)=1+sin(πt)103+u20+|v|100≤1.221≤Mc≈1.772

for (*t*, *u*, *u*′) ∈ [0,1]×[0,4]×[−4,4]. Hence, all the conditions of Theorem 17 are satisfied. By Theorem 17, BVP (55) has at least three positive solutions *u*_1_, *u*_2_, and *u*_3_ such that

(60) $$\begin{array}{c} \underset{t\in \left[0,1\right]}{\max}\left|u_{1}\left(t\right)\right|<\frac{1}{100},\\ \frac{1}{50}<\underset{t\in \left[0,1/6\right]}{\min}\left|u_{2}\left(t\right)\right|<\underset{t\in \left[0,1\right]}{\max}\left|u_{2}\left(t\right)\right|\leq 4,\\ \frac{1}{100}<\underset{t\in \left[0,1\right]}{\max}\left|u_{3}\left(t\right)\right|\leq 4,\;\;\underset{t\in \left[0,1/6\right]}{\min}\left|u_{3}\left(t\right)\right|<\frac{1}{50}. \end{array}$$

**Example 3**

Consider the following fractional BVP:

(61) D0+5/2Cu(t)=f(t,u(t),u′(t)),   0<t<1,u(1)=u′(1)=u′′(0)=0,

where

(62) $$\begin{array}{c} f\left(t,u,v\right)=\frac{t}{40}+6u^{3}+\frac{{\left|u^{\prime }\right|}^{1/6}}{3^{10}}. \end{array}$$

After a simple computation, one can find that

(63) M′=43π≈0.752445,  N′≈0.03,Q=83π≈1.50489.

Choosing *d* = 36, *q* = 1/3, and *L* = 5^9^ = 1953125, we know that

(64) $$\begin{array}{c} f\left(t,u,u^{\prime }\right)\leq 0.248<\frac{q}{M^{\prime }}\approx 0.443 \end{array}$$

for (*t*, *u*, *u*′) ∈ [0,1]×[0, 1/3]×[−5^9^, 5^9^];

(65) $$\begin{array}{c} f\left(t,u,u^{\prime }\right)\geq 1296\geq \frac{d}{N^{\prime }}\approx 1200 \end{array}$$

for (*t*, *u*, *u*′) ∈ [0, 1/6]×[6,36]×[−5^9^, 5^9^]; and

(66) $$\begin{array}{c} f\left(t,u,u^{\prime }\right)\leq 279936.03<\frac{L}{Q}\approx 1302083.3 \end{array}$$

for (*t*, *u*, *u*′) ∈ [0,1]×[0,36]×[−5^9^, 5^9^]. So, all the conditions of Theorem 18 are satisfied. By Theorem 18, BVP (61) has at least one positive solution *u*(*t*) satisfying 1/3 < max⁡_*t*∈[0,1]_⁡|*u*(*t*)| < 36, |*u*′(*t*)| < 5^9^.

## 5. Conclusion

In this paper, we study the existence of multiple positive solutions for the nonlinear fractional differential equation boundary value problem (1) in the Caputo sense. Using Guo-Krasnoselskii and Leggett-Williams fixed point theorems, we establish the existence of multiple positive solutions to BVP (1). By virtue of a new extension of Krasnoselskii's fixed point theorem, we obtain a sufficient condition for the existence of multiple positive solutions of BVP (1) from its auxiliary problem. As applications, examples are presented to demonstrate the main results.

## References

[1] Atangana A, Secer A (2013). A note on fractional order derivatives and table of fractional derivatives of some special functions. *Abstract and Applied Analysis*.

[2] Atangana A, Secer A (2013). Time-fractional coupled-Korteweg-de-Vries equations. *Abstract and Applied Analysis*.

[3] Bai C-Z, Fang J-X (2004). The existence of a positive solution for a singular coupled system of nonlinear fractional differential equations. *Applied Mathematics and Computation*.

[4] Cang J, Tan Y, Xu H, Liao S-J (2009). Series solutions of non-linear Riccati differential equations with fractional order. *Chaos, Solitons and Fractals*.

[5] Girejko E, Mozyrska D, Wyrwas M (2011). A sufficient condition of viability for fractional differential equations with the Caputo derivative. *Journal of Mathematical Analysis and Applications*.

[6] Li CF, Luo XN, Zhou Y (2010). Existence of positive solutions of the boundary value problem for nonlinear fractional differential equations. *Computers & Mathematics with Applications*.

[7] Wei Z, Li Q, Che J (2010). Initial value problems for fractional differential equations involving Riemann-Liouville sequential fractional derivative. *Journal of Mathematical Analysis and Applications*.

[8] Yang X, Wei Z, Dong W (2012). Existence of positive solutions for the boundary value problem of nonlinear fractional differential equations. *Communications in Nonlinear Science and Numerical Simulation*.

[9] Liang S, Zhang J (2009). Positive solutions for boundary value problems of nonlinear fractional differential equation. *Nonlinear Analysis, Theory, Methods and Applications*.

[10] Zhang S, Su X (2011). The existence of a solution for a fractional differential equation with nonlinear boundary conditions considered using upper and lower solutions in reverse order. *Computers & Mathematics with Applications*.

[11] Zhao Y, Sun S, Han Z, Li Q (2011). The existence of multiple positive solutions for boundary value problems of nonlinear fractional differential equations. *Communications in Nonlinear Science and Numerical Simulation*.

[12] Kexue L, Jigen P (2011). Laplace transform and fractional differential equations. *Applied Mathematics Letters*.

[13] Podlubny I (1994). *The Laplace Transform Method for Linear Differential Equations of Fractional Order*.

[14] Samko G, Kilbas A, Marichev O (1993). *Fractional Integrals and Derivatives: Theory and Applications*.

[15] Miller KS, Ross B (1993). *An Introduction to the Fractional Calculus and Fractional Differential Equations*.

[16] Jafari H, Seifi S (2009). Solving a system of nonlinear fractional partial differential equations using homotopy analysis method. *Communications in Nonlinear Science and Numerical Simulation*.

[17] Zurigat M, Momani S, Odibat Z, Alawneh A (2010). The homotopy analysis method for handling systems of fractional differential equations. *Applied Mathematical Modelling*.

[18] Chai G (2011). Existence results for boundary value problems of nonlinear fractional differential equations. *Computers & Mathematics with Applications*.

[19] Liu Y, Zhang W, Liu X (2012). A sufficient condition for the existence of a positive solution for a nonlinear fractional differential equation with the Riemann-Liouville derivative. *Applied Mathematics Letters*.

[20] Xu X, Fei X (2012). The positive properties of Green’s function for three point boundary value problems of nonlinear fractional differential equations and its applications. *Communications in Nonlinear Science and Numerical Simulation*.

[21] Bai Z, Qiu T (2009). Existence of positive solution for singular fractional differential equation. *Applied Mathematics and Computation*.

[22] Krasnosel’skii MA (1964). *Positive Solutions of Operator Equations*.

[23] Leggett RW, Williams LR (1979). Multiple positive fixed points of nonlinear operators onordered Banach spaces. *Indiana University Mathematics Journal*.

[24] Guo Y, Ge W (2004). Positive solutions for three-point boundary value problems with dependence on the first order derivative. *Journal of Mathematical Analysis and Applications*.

